# Determination of the Environmental Pollution Potential of Some Herbicides by the Assessment of Cytotoxic and Genotoxic Effects on *Allium cepa*

**DOI:** 10.3390/ijerph16010075

**Published:** 2018-12-28

**Authors:** Catalin Aurelian Rosculete, Elena Bonciu, Elena Rosculete, Liviu Aurel Olaru

**Affiliations:** 1 Department of Land Measurement, Management, Mechanization, Faculty of Agronomy, University of Craiova, 13 A.I. Cuza Street, 200585 Craiova, Romania; catalin_rosculete@yahoo.com; 2 Department of Agricultural and Forestry Technology, Faculty of Agronomy, University of Craiova, 13 A.I. Cuza Street, 200585 Craiova, Romania; liviu.olaru.dtas@gmail.com

**Keywords:** *Allium cepa*, herbicides, mitotic index, chromosomal aberrations, cytotoxicity, genotoxicity, pollution potential

## Abstract

The present study aims to evaluate the potential for the pollution of the environment by two herbicides (quizalofop-p-ethyl and cycloxydim), using the *Allium* test. The species in question is *Allium cepa* (onion, 2*n* = 16), one of the most common plant indicators of environmental pollution. The working method consisted of obtaining the meristematic roots of *Allium cepa* and their treatment with herbicides at three different concentrations (0.5%, 1%, and 1.5%) for each herbicide for 24 h, for comparison with an untreated control. The results obtained from the cytological study indicated a strong cytotoxic and genotoxic effect for both herbicides, but especially for quizalofop-p-ethyl, where the mitotic index decreased from 30.2% (control) to 9.6% for the variant treated with 1.5% herbicide. In this case, a strong mitodepressive effect was shown by a highly significant percentage (35.4%) of chromosomal aberrations and nuclear alterations: stickiness, fragments, C-mitosis, lobulated nucleus, micronuclei, and nuclear erosion. The mitodepressive effect as well as the percentage of chromosomal aberrations increased with a higher herbicide concentration. The obtained results suggest the strong potential for pollution of the two herbicides, particularly at concentrations higher than 0.5%; therefore, we recommend caution in their use to avoid undesirable effects on the environment.

## 1. Introduction

Agriculture, in conjunction with industry, is a major source of pollutant agents, with a negative impact on the quality of the environment. Herbicides are chemical weed control chemicals, representing a revolution in plant breeding technology. Using them excessively can cause serious damage to the environment [1,2,3], and also to the people exposed to their actions [4].

The handling and use of herbicides must follow all appropriate precautionary procedures, because there is a close correlation between diseases, especially cancer, and occupational exposure to these chemical compounds [5].

In Romania in 2013, the herbicide-treated area held the largest share of the pesticide-treated surfaces, both in the solid and in liquid form. In terms of the quantity used in the liquid form, herbicides hold the largest share in the total plant protection products used (62.0%), followed by fungicides (27.0%), and insecticides (9.6%) [6].

Quizalofop-p-ethyl and cycloxydim are liquid herbicides belonging to the arylphenoxy propionate group, used in post-emergence to control annual and perennial monocotyledonous weeds in sunflower crops, maize, rape, potato, and tomato. 

Quizalofop-p-ethyl and cycloxydim are selective, post-emergence herbicides approved for use in the EU. They are soluble in aqueous solutions, are relatively volatile, have a broad spectrum, and are widely used to control annual and perennial grasses in a large variety of broad-leaved crop plants.

Currently, mainstream literature provides few data regarding quizalofop-p-ethyl phytotoxicity. In vitro pollen germination and tube growth of *Hyacinthus orientalis* were affected by quizalofop-p-ethyl treatments and caused changes in the morphological features to this species [7]. The amount of leaf pigments of *Glycine max* as well as the root and seedling length were significantly affected by quizalofop-p-ethyl treatment, at a concentration of 0.8 M [8]. The oral treatment of quizalofop-p-ethyl in female albino Wistar rats produced significant harmful effects on some haematological and biochemical parameters. This herbicide is found to be harmful if aspirated, because it may cause lung damage [9]. Therefore some authors recommend to avoid recurrence in order to minimize risks to persons using the product in the workplace [9,10].

In some experiments investigating the general pharmacological effects of quizalofop-ethyl, a slight inhibition of the central nervous system was observed in mice (reduced motor activity, retarded pinna reflex, and hypothermia). It was presumed that quizalofop-ethyl might possess a small inhibitory effect on the central nervous system in mice [11]. According to the European Food Safety Authority (EFSA) Scientific Report (2008), the risk to aquatic organisms was assessed to be low. 

Cycloxydim belongs to the cyclohexanedione class. Cyclohexanedione herbicides are known to be biologically active at very low concentrations. Their polar character makes them easily leach into groundwater and potentially contaminate at levels above 0.1 μg L^−1^. Cycloxydim exhibits a high to very high mobility in soil and can potentially contaminate groundwater. The acute toxicity data carried out by the EFSA (2010) indicated that technical cycloxydim is harmful to aquatic organisms [12]. Cycloxydim showed a significant inhibitory effect on the population density of the freshwater ciliate *Paramecium tetraurelia* in a concentration-dependent manner, and the growth rate was reduced significantly with the increase of herbicide concentrations [13].

Plants are the main components of a healthy environment since they produce oxygen and organic carbon and this is the reason why many plant species are considered indicators of unfavourable environmental factors [14]. *Allium cepa* is one of the most frequently used higher plant species for cytotoxicity and genotoxicity assays of various environmental pollutants [15,16]. Also, the *Allium* test enables the assessment of different genetic endpoints causing damage to the DNA of humans [17]. 

In addition, other plants have also been employed as models for investigating the cytotoxicity and genotoxicity of chemical compounds and environmental pollutants tests described for *Allium cepa*; among these, the *Lactuca sativa* L. (lettuce) model can be highlighted [18].

*Allium cepa* is more sensitive than other tests when detecting toxicity and genotoxicity; this application plays an important role in bio-monitoring due to the fact that roots of onions are sensitive to any toxic materials [19]. Therefore, we used this test in our work to evaluate the environmental pollution potential of the quizalofop-p-ethyl and cycloxydim herbicides.

The aim of study was to explore the cytotoxic and genotoxic effects of these herbicides on *Allium cepa* by observing and interpreting the decreased mitotic index and occurrence of the chromosomal aberrations. We chose this topic because there are many small-sized family farms in Romania, where the manufacturer’s indications for the application of any pesticides (herbicides, insecticides and fungicides) are probably not always followed in terms of the recommended doses.

### Theory—Chemistry and Toxicology of Quizalofop-P-Ethyl and Cycloxydim

Quizalofop-p-ethyl is the International Organization for Standardization (ISO) common name for ethyl (2R)-2-[4-(6-chloroquinoxalin-2-yloxy)phenoxy] propionate (IUPAC-International Union of Pure and Applied Chemistry). It is an ester variant of quizalofop-p. Quizalofop-p-ethyl belongs to the class of aryloxyphenoxy propionic herbicides (commonly called ‘FOPs’). It is a selective, post-emergence herbicide that is used to control annual and perennial weeds in various crops.

**Figure 1:**
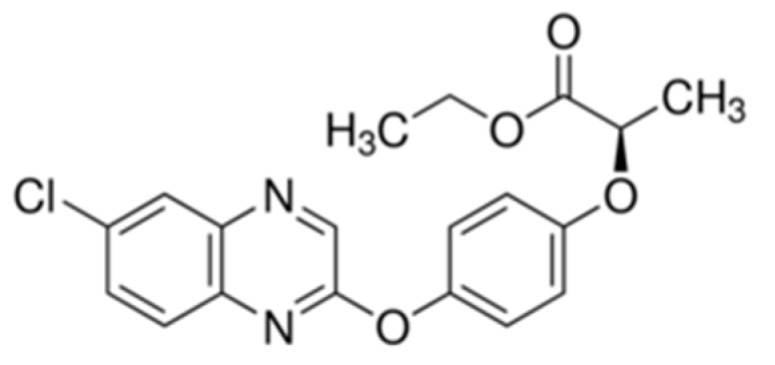
Structure of quizalofop-p-ethyl. Molecular mass: 372.8 g/mol.

Quizalofop-p-ethyl is absorbed from the roots and leaf surface and is moved throughout the plant. The ester is hydrolysed in the plant to free acid which is the actual active form. The mode of action is by the inhibition of lipid biosynthesis in target plants [20]. The chemical structure of the compound is reported below (Figure 1). 

The metabolism of quizalofop-p-ethyl in plants has been studied in roots and tuber vegetables (potato, sugar beet) and in pulses and oilseeds (cotton, soya, bean) [20]. The parent ester has not been generally detected or identified in low portions in mature plant parts at harvest, except in beet leaves (20% of the total radioactive residue (TRR)) and in soya straw (47% of the TRR). The high portion of quizalofop-p-ethyl in soya straw detected in one study was, however, not confirmed in two other studies conducted under similar treatment conditions. The major metabolite was quizalofop, which was always present at harvest and conjugates of quizalofop and quizalofop-phenol, which were always present in soya beans and straw (15–33% TRR). The other identified metabolites were generally present in low levels (<10% of the TRR) with the exception of the phenoxy propionate, which accounted for 16% of the TRR (0.07 mg/kg) in sugar beet leaves, 92 days after the quizalofop-p-ethyl application [20].

According to the results from all available metabolism studies in primary and rotational crops, once quizalofop is formed after the hydrolysis of the ester link, the metabolic pathways of the different esters in plants are similar. The parent ester is rapidly degraded to quizalofop, which, together with its conjugates was always present at harvest [20].

For certain commodities, the available residue trials were not sufficient to derive risk assessment values for the use of all the variants, and it could not be excluded that those uses not supported by data would result in higher residue levels, in particular when the existing EU maximum residue level (MRL) is higher than in the MRL proposal. In these cases, the EFSA decided, adopting a conservative approach, to use the existing EU MRL for an indicative exposure calculation. All input values refer to the residues in the raw agricultural commodities [20]. It is noted that more critical Good Agricultural Practices (GAPs) not supported by data are authorized for quizalofop-p-ethyl in northern European countries in the case of potatoes, beetroots, carrots, celeriacs, parsnips, salsifies, spinach, parsley, beans with pods, and fruit spices, and in southern European countries for apples, pears, loquats, apricots, cherries, peaches, plums, table and wine grapes, strawberries, potatoes, head cabbage, lettuce, escaroles, peas without pods, fresh lentils, sunflower seeds, rapeseeds, soybeans, and sugar beets [20].

Cycloxydim is a systemic foliar herbicide. The active substance is used to control annual and perennial grass weeds as well as volunteer cereals in broadleaf crops such as sugar beets, oilseed rape, potatoes, beans and cycloxydim-tolerant maize. After uptake via the aerial parts of the plants it acts as an inhibitor of acetyl-CoA-carboxylase in susceptible species [21].

**Figure 2:**
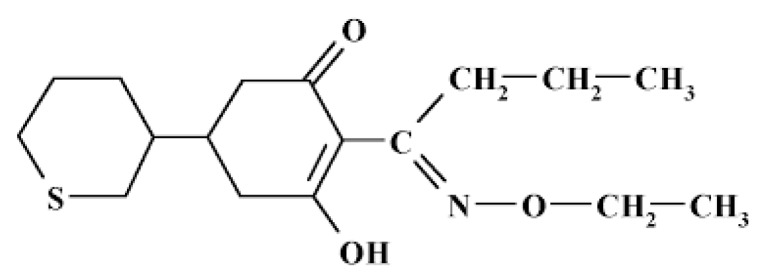
Structure of cycloxydim. Molecular mass: 325.5 g/mol.

Cycloxydim is the ISO common name of (5RS)-2-[(EZ)-1(ethoxyimino) butyl]-3-hydroxy-5-[(3RS)-thian-3-yl]cyclohex-2-en-1-one (IUPAC). Cycloxydim is racemic, with a chiral centre in the heterocycle, whereas the carbocycle is essentially symmetric due to the facile tautomery of the vinologous acid. The ratio of E:Z in technical material is of 99.2:0.8; the ratio R:S is 1:1 (racemic mixture) [21]. The chemical structure of the compound is reported below (Figure 2). 

The toxicological profile of cycloxydim was assessed in the framework of the peer review under Directive 91/414/EEC, and the data were sufficient to derive an acceptable daily intake (ADI) of 0.07 mg/kg body weight (bw) and an acute reference dose (ARfD) of 2 mg/kg bw. The metabolism of cycloxydim in primary crops was investigated in the root (sugar beet), pulses/oilseeds (soybean) and cereals (maize) crop groups, using a single foliar application [21]. The EFSA report (2015) concluded that the intended use of cycloxydim on beetroots, celeriac, parsnips, horse radishes, Jerusalem artichokes, swedes, aubergines, Brussels sprouts, head cabbage, kale, Chinese cabbage, scarole, spinach, beet leaves, oilseed rape, and roots of herbal infusions would not result in a consumer exposure exceeding the toxicological reference values and therefore it is unlikely to raise public health concerns [21].

The occurrence of cycloxydim residues in rotational crops was investigated in the framework of peer review. Based on the available information, it was concluded that significant residues of cycloxydim were unlikely to occur in rotational crops [21].

The improvement of the systems and methods for weed control requires a continuous search and the introduction of new herbicides, whose effect complies with the contemporary agro–ecological conditions [22,23,24].

## 2. Materials and Methods 

### 2.1. Plant Material 

Healthy and equal-sized bulbs (30–35 mm in diameter) of a commercial variety of onion (*Allium cepa*, 2*n* = 16) made up the experimental material. The dried leaves and roots were removed, after which the experimental material was transferred to small glass bottles with tap water (the germinal disc being immersed in liquid) to enable meristematic roots to grow. After 72 h, the *Allium cepa* bulbs were transferred to another small glass bottles containing the herbicides treatment solutions: quizalofop-p-ethyl and cycloxydim in concentrations of 0.5%, 1%, and 1.5% for each of them. Herbicides were purchased in 500-mL bottles from a phytopharmaceutical shop in the Craiova city, Romania.

The concentration recommended by the manufacturers for both herbicides is 0.5%, therefore, for the *Allium* test, this value was the first choice. Concentrations of 1% and 1.5%, respectively, are an extrapolation of the recommended concentration and were chosen on account of the fact that many farmers may try to potentiate the action of herbicides by using a lower dilution.

The bulbs were kept with the meristematic roots immersed in these solutions for 24 h. We considered that the 24-h exposure time was necessary for the active substance of the herbicide to be completely absorbed by the *Allium cepa* roots.

The control variant was further maintained in plain water. Afterwards, the meristematic roots were cut and processed for microscopic preparation. Prior to the microscopic preparation step, the meristematic roots of each variant, including those of the control variant, were measured to assess the potential of each tested herbicide to inhibit the growth of roots.

### 2.2. Microscopic Preparations

The biological material was fixed with a mixture of absolute ethyl alcohol and glacial acetic acid in a volume ratio of 3:1 at 6 °C in the refrigerator for 24 h, followed by hydrolysis with 1 N hydrochloric acid at room temperature for 5 min. The stage of the meristematic roots staining was performed using the Feulgen–Rossenbeck method. Colouring was achieved in a basic fuchsine solution, with a concentration of 10%. The staining time was of 90 min, followed by the intensification of the coloration in plain water for 20 min.

The microscopic slides were prepared using the squash technique. Five slides and 500 cells for each variant were analysed in order to calculate the mitotic index and the chromosomal aberration frequency. The same slides used to calculate the mitotic index were studied to identify the chromosomal aberration. All slides were examined using an optical microscope with in-built digital camera.

The study evaluated mitotic aberrations (stickiness, fragments, C-mitosis) and nuclear anomalies (lobulated nuclei, micronuclei and cells with nuclear erosion).

### 2.3. Statistical Analyses

Statistical analysis was carried out using MS Excel 2007. The analysis of variance (ANOVA) was used to assess the significant differences between the control variant and each treatment. The data were expressed as mean ± standard error of mean (SEM). The SEM was calculated for the mitotic index (MI) and for total abnormalities (TA), comprising both chromosomal aberrations and nuclear anomalies. The differences between treatment means were compared using the Least Significant Difference (LSD) test at a probability level of 0.05% subsequent to the ANOVA analysis.

The mitotic index was calculated using the following formula:

$$\mathrm{MI}\;(\%)=\frac{\mathrm{Total}\;\mathrm{number}\;\mathrm{of}\;\mathrm{cells}\;\mathrm{in}\;\mathrm{division}}{\mathrm{Total}\;\mathrm{number}\;\mathrm{of}\;\mathrm{analysed}\;\mathrm{cells}}\times 100$$

The index of the total abnormalities (TAs) was also calculated:

$$\mathrm{TA}\;(\%)=\frac{\mathrm{Total}\;\mathrm{number}\;\mathrm{of}\;\mathrm{aberrant}\;\mathrm{cells}}{\mathrm{Total}\;\mathrm{number}\;\mathrm{of}\;\mathrm{cells}\;\mathrm{in}\;\mathrm{division}}\times 100$$

## 3. Results

**Figure 3:**
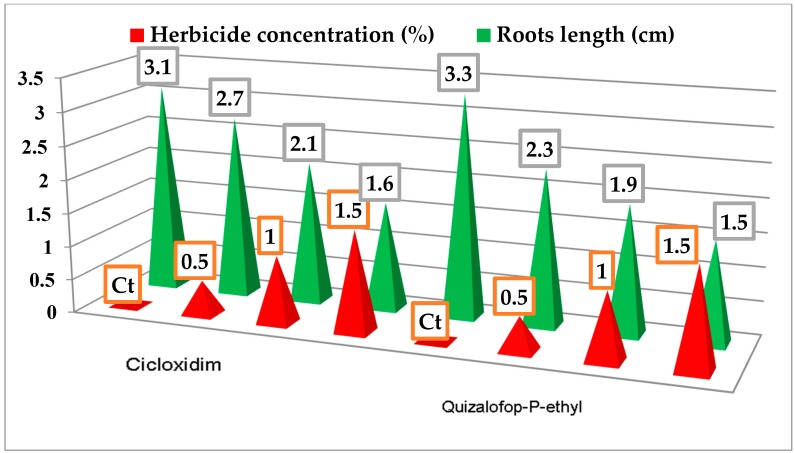
The inhibitory effect of cycloxydim and quizalofop-p-ethyl herbicides on the growth of the *Allium cepa* roots.

The effects of cycloxydim and quizalofop-p-ethyl herbicides on the growth of the *Allium cepa* roots are shown in Figure 3. Thus, the maximum root growth was observed on the control variant in relation to both herbicides (3.1 and 3.3 cm, respectively). As the concentration of the two herbicides increased the effect of inhibiting root growth increased from 2.7 cm to 1.6 cm (cycloxydim) and from 2.3 cm to 1.5 cm (quizalofop-p-ethyl). It can be seen that the herbicide quizalofop-p-ethyl showed a larger potential for the growth inhibition of *Allium cepa* roots compared to cycloxydim.

From the point of view of the cytotoxicity effect induced by the two types of herbicides to *Allium cepa*, the results indicate that the MI decreased in all variants with increased herbicide concentration (Table 1). Thus, in the case of the cycloxydim herbicide, the MI recorded values amounted to 34.1% (Ct), 28.1% (V1), 24.2 (V2) and 15.9 (V3), whereas the quizalofop-p-ethyl treatment was more cytotoxic, the MI registering significantly lower values compared to the control variant: 19.8% (V1), 11.9% (V2) and 9.6% (V3), respectively. 

On the other hand, the prophase, metaphase, anaphase and telophase of the mitotic division recorded lower values compared to the control variant, as the herbicide concentration increased, while the interphase (which is the stage of mitotic division preparation) increased. In this case, too, there is a larger cytotoxic potential of the herbicide quizalofop-p-ethyl.

**Figure 4:**
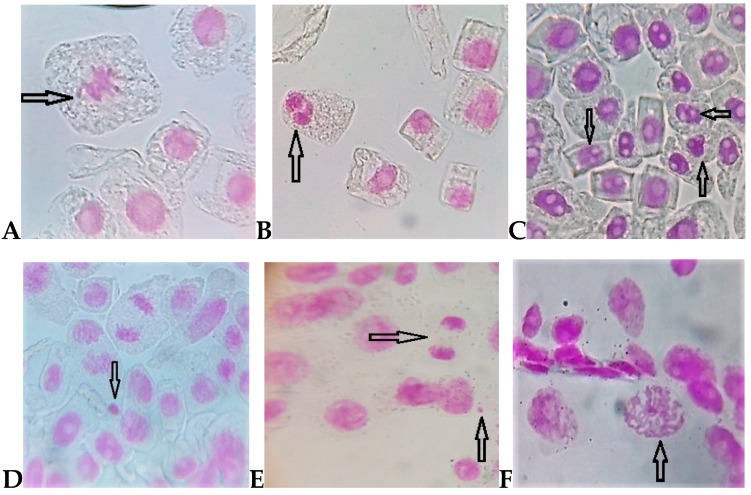
Some chromosomal aberrations and nuclear anomalies identified in meristematic cells of *Allium cepa* exposed to cycloxydim and quizalofop-p-ethyl herbicides: Fragments in metaphase (**A**); nuclear erosion (**B**); cells with lobulated nucleus (**C**); micronucleus (**D**); telophase stickiness and micronucleus (**E**); sticky chained metaphase in a polyploid cell (**F**).

The tested herbicides induced a high number of mitotic aberrations and nuclear anomalies in the cells of *Allium cepa*: stickiness, fragments, C-mitosis, lobulated nuclei, micronuclei, and cells with nuclear erosion (Figure 4). The increase of TA was dependent on increasing herbicides concentrations (Table 2). The stronger genotoxic potential was registered by quizalofop-p-ethyl 1.5% (V3), where the TA percentage was of 35.4%, significantly higher than the control variant. But at a concentration of 1.5%, the cycloxydim herbicide also triggered a distinct significantly higher genotoxic effect compared to the control variant, accounting for a total of 23.3% of TA in *Allium cepa* meristematic cells.

## 4. Discussion

Plant bioassays prove to be efficient tests for the genotoxicity monitoring of environmental pollutants [25,26,27]. *Allium* test is a standard test for rapid and sensitive screening of pollutants that represent environmental hazards. The use of *Allium cepa* for the bio-monitoring of genotoxicity is considered to be highly efficient by several authors [28,29]. Employing the *Allium cepa* as a test system to detect mutagens dates back to the 1940s, and it has been used to assess a high number of chemical agents, which contributes to its widespread application in environmental monitoring [30].

The root tip system of *Allium cepa* has particularly shown sensitivity to the harmful effects of environmental hazards [31]. By analysing the results obtained, it was noticed that both herbicides inhibited the meristematic root growth of *Allium cepa* compared to the control variant, although the herbicide quizalofop-p-ethyl displayed larger toxicity potential. The root growth decrease as well as the types and frequencies of chromosome aberrations are an indicator of the toxic effects of environmental pollutants [32,33]. 

MI is considered to reliably identify the presence of cytotoxic pollutants in the environment [34]. As shown in mainstream literature, the decrease of the mitotic index value below 50% compared to the control variant leads to a sublethal effect, while below 22% it can cause lethal effects on test organisms [3,35,36]. Admittedly, at a concentration of 1.5% (V3), the cycloxydim herbicide triggered a sublethal cytotoxicity effect on *Allium cepa*, while the quizalofop-p-ethyl herbicide had a potential for sublethal effects even at a concentration of 1%. The mitodepressive effect of the two herbicides described their cytotoxic and pollutant potential in *Allium cepa* meristematic roots. Through the *Allium* test, other authors reported similar results about the cytotoxic and genotoxic potential of some herbicides to plants. Avenoxan significantly induced abnormalities such as C-mitosis, chromosome stickiness, bridges, laggards, multipolar cells, and significantly decreased mitotic index in both *A. cepa* L. and *Allium sativum* L. [37].

In another study [2], Illoxan showed a mitodepressive effect and caused clastogenic and aneugenic types of abnormalities in *Allium cepa* root tip cells. Furthermore, some authors [38] indicated that the herbicide imazethapyr exhibited cytotoxic activity but no genotoxic activity (except 10 ppm) and caused DNA damage in *Allium cepa* root meristematic cells.

In our study, the frequency of cells with sticky chromosomes increased with increasing cycloxydim and quizalofop-p-ethyl concentrations. Actually, stickiness was the most frequent chromosomal aberration observed in root tips of *Allium cepa* treated with both types of herbicides. On the contrary, no sticky chromosomes were observed in the control variant. Other researchers suggest that sticky chromosomes reflect highly toxic effects and probably lead to cell death [39]. Genotoxicity is one of the serious side effects of pesticide exposure [40].

Another aberration induced by the cycloxydim and quizalofop-p-ethyl herbicide in the meristematic cells of *Allium cepa* was the lobulated nucleus, indicating disturbance in the synthesis of nucleic acids. These results comply with those reported by [41] who conclude that the presence of lobulated nuclei reveal a cell death process.

C-mitosis is the result of damaged mitotic apparatus due to genotoxic substances in the cells, and it is stimulated by many chemicals [34,42]. In our study, these types of chromosomal aberrations had a frequency of 4.3–5.5% in the case of the cycloxydim treatment, while in the case of quizalofop-p-ethyl the frequency of C-mitosis was of 3.1–8.2%; these results signal the genotoxic and pollutant potential of these herbicides to plants and the environment alike. However, pollution varies with the change of the spatial position, and the pollution of the soil determined through statistical analyses of solely the sampling points cannot reflect the spatial distribution characteristics of the whole study area [43]. The environmental risk of pollutants is influenced by their absorption behaviour [44]. 

## 5. Conclusions

Agricultural activities cause environmental pressures that can be even more damaging if farming practices are incorrectly applied. One of these practices is the excessive use of herbicides, but probably also of other pesticides, and therefore it requires environmental assessment and monitoring measures.

The mitodepressive effect and the occurrence of a large number of chromosome aberrations and nuclear abnormalities in *Allium cepa* following the testing of the herbicide cycloxydim and quizalofop-p-ethyl suggest their high environmental pollution potential.

We intend to continue testing them (through the *Allium* test) on a large area of agricultural land, where farmers often excessively apply both herbicides and other pesticides. We would like to raise awareness of the pesticide users regarding the risks they pose to the environment, as well as regarding their own exposure to these. It must be kept in mind that the state of the environment influences, in addition to the conditions of economic growth, the level and quality of life.

## Figures and Tables

**Table 1:** Mitotic index and mitotic phases of *Allium cepa* meristematic roots treated with different doses of herbicides.

Herbicide/Exposure Time (h)	Variants/Concentration (%)	MI ± SEM (%)	Mitotic Phases				
			I (%)	P (%)	M (%)	A (%)	T (%)
Cycloxydim/24	Control	34.1 ± 1.9	13.4	49.1	12.4	10.3	14.8
	V1/0.5	28.1 ± 0.5	19.1	45.2	12.0	9.6	14.1
	V2/1.0	24.2 ± 0.2 *	30.4	42.6	8.3	6.1	12.6
	V3/1.5	15.9 ± 0.3 **	51.4	34.8	2.6	2.0	9.2
Quizalofop-p-ethyl/24	Control	30.2 ± 1.8	17.1	42.4	14.1	11.0	15.4
	V1/0.5	19.8 ± 1.3 **	30.8	40.7	8.4	6.9	13.2
	V2/1.0	11.9 ± 0.3 ***	52.6	30.5	5.8	3.6	7.5
	V3/1.5	9.6 ± 0.7 ***	65.6	26.2	2.4	1.5	4.3

MI = mitotic index; SEM = standard error of mean; I = interphase; P = prophase; M = metaphase; A = anaphase; T = telophase. The results are expressed as the mean ± SEM; * significant at *p* ≤ 0.05, ** significant at *p* ≤ 0.01, *** significant at *p* ≤ 0.001 as compared to the control variant Least Significant Difference (LSD) test at a probability level of 0.05% subsequent to the ANOVA analysis).

**Table 2:** Type and percentage of mitotic aberrations and nuclear abnormalities induced by some herbicides in the cells of *A. cepa*.

Herbicide/Exposure Time (h)	Concentration (%)	Mitotic Aberrations and Nuclear Abnormalities (%)						TA (%)
		S	F	CM	LN	MN	NE	
Cycloxydim/24	Control	0.4	0	1	0	0	0	1.4
	V1/0.5	4.3	0.6	4.3	0	0.5	2.7	12.4
	V2/1.0	4.9	1.9	5.5	1.2	1.7	4.1	19.3 *
	V3/1.5	5.4	3.0	4.8	2.6	4.3	3.2	23.3 **
Quizalofop-P-ethyl/24	Control	0.5	0	1.5	0	0	0	2.0
	V1/0.5	4.4	3.6	3.1	1.3	1.7	3.1	17.2 *
	V2/1.0	6.8	2.2	6.1	1.6	4.6	2.8	24.1 **
	V3/1.5	7.5	3.9	8.2	3.7	6.3	5.8	35.4 ***

TA = total abnormalities; S = stickiness; F = fragments; CM = C-mitosis; LN = lobulated nucleus; MN = micronuclei; NE = nuclear erosion. The results are expressed as the mean ± SEM; * significant at *p* ≤ 0.05, ** significant at *p* ≤ 0.01, *** significant at *p* ≤ 0.001 compared to the control variant (LSD test at a probability level of 0.05% subsequent to the ANOVA analysis).
